# Rare case of combined vascular malformation of the face: a successful surgical management

**DOI:** 10.1308/rcsann.2022.0073

**Published:** 2024-05-30

**Authors:** S Bhatt, RM Karmacharya, S Vaidya, D Prajapati, R Napit, K Chapagain, N Bhandari, A Tamang

**Affiliations:** Kathmandu University School of Medical Sciences, Dhulikhel Hospital, Nepal

**Keywords:** Case report, Facial asymmetry, Masseter muscle, Sclerotherapy, Vascular malformations

## Abstract

Combined vascular malformation affecting the facial region is an extremely rare clinical entity that is debilitating both functionally and emotionally. Treatment warrants a multidisciplinary approach with the aim of removing the vascular anomalies and ameliorating any functional facial disfigurement. Here, we present a case of a 40-year-old female with combined vascular malformation of the face who was treated successfully with surgical intervention accompanying significant resolution of facial disfigurement.

## Background

Vascular malformations are subgrouped under capillary, venous, arterial, lymphatic and combined forms.^1^ These anomalies affecting the facial region are uncommon and can be significantly debilitating with psychological disturbance owing to functional facial disfigurement.^2^ However, there is a paucity of literature revealing the prevalence and management of the combined type of malformation.

## Case history

**Figure 1:**
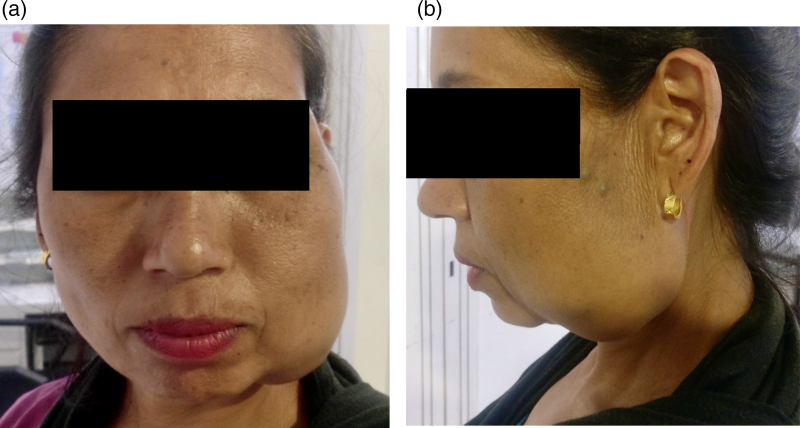
Enlarged left side of the face: (a) frontal view, (b) lateral view.

**Figure 2:**
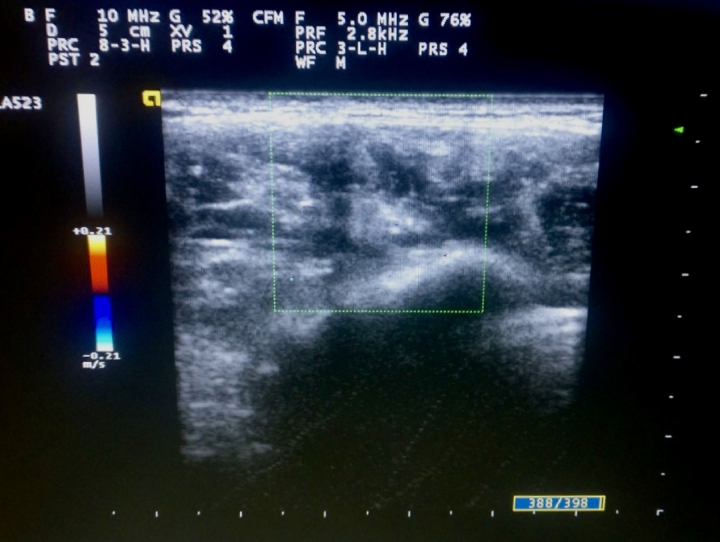
Ultrasonography showing combined vascular malformation with predominant venous malformation along with some arterial and lymphatic components

A 40-year-old female presented with chief complaints of progressively increasing swelling in the left jaw region over a period of more than 6 years (Figure 1). The swelling was associated with occasional pain that was aggravated during the winter and difficulty in chewing. In addition, the patient had a major cosmetic concern. She denied the presence of swelling since early childhood, history of trauma, surgery, pregnancy or use of hormonal contraceptives. On clinical examination, a large swelling measuring 8 × 6cm extending from the left lower jaw to the mid-jaw anteriorly and to the mid-cheek region superiorly was found. The swelling was hard, non-compressible, non-fluctuant, non-tender, non-pulsatile and immobile, with no overlying skin and temperature changes or intraoral extension. On auscultation, bruit was not heard. Ultrasonography of the overlying swelling was done, which revealed a combined vascular malformation measuring 7 × 5cm with a venous predominance (Figure 2). In addition, a positive turkey wattle sign was noted.

**Figure 3:**
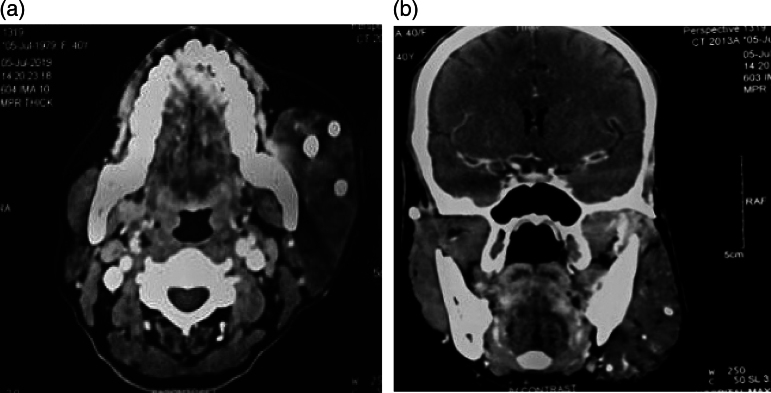
Multidetector computed tomography of the neck with intravenous contrast: (a) axial view and (b) coronal Multiplanar Reconstruction (MPR) view showing combined vascular malformation of size 7.7 × 5.2cm with both venous and arterial components.

Plain and contrast-enhanced multidetector computed tomography (MDCT) of the neck was done and revealed the presence of a 7.7 × 5.2cm lesion with venous predominance along with an arterial component (Figure 3). In addition, multiple phleboliths extending to the left temporalis and pterygoid muscle, predominantly in the left masseter muscle, were present. All findings were suggestive of combined vascular malformation of the face.

An intralesional steroid injection using 40mg triamcinolone was administered along with sclerotherapy without coiling to decrease the flow in the lesion. After one week, minimal resolution of the lesion was noted and surgical intervention was therefore planned.

**Figure 4:**
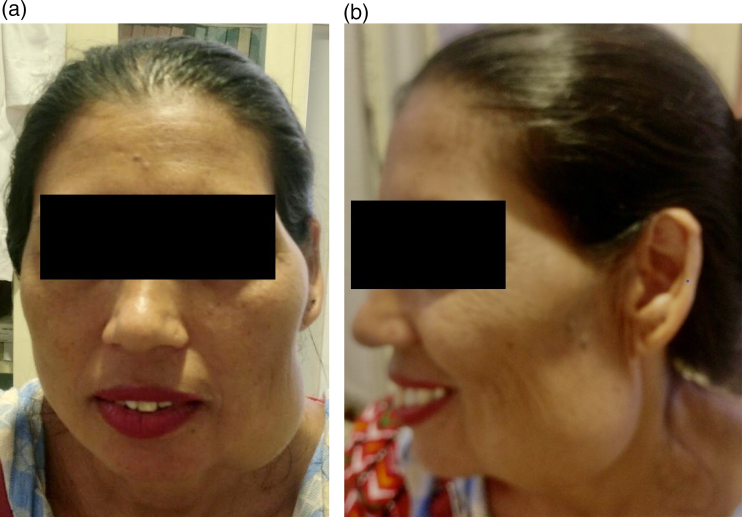
Patient outcome following administration of intralesional steroid on the face: (a) frontal view (b) lateral view.

Intraoperative findings revealed the presence of arteriovenous malformation over the left temporalis and pterygoid muscles; extending from mid-jaw anteriorly, lateral pterygoid muscle posteriorly, the mid-part of the temporalis muscle superiorly and the angle of the jaw inferiorly. Ligation of the feeders and significant removal of vascular malformation was done with the maintenance of proper hemostasis and preservation of all the muscles of mastication. However, vascular malformation in the intramuscular part was not removed because it would have hampered the patient’s ability to chew. For this, she underwent sclerotherapy of the remaining venous channels during surgery. On the first postoperative day, Approximately 500 ml of blood was transfused owing to blood loss, after which her haemoglobin remained above 10 g/dl. Ceftriaxone 1g and flucoxacillin 500mg were used as antibiotic prophylaxis, with a five-day hospital stay. One month following the surgery, there was a marked reduction in the size of the lesion. Seroma, which formed postoperatively in the lower part of the jaw, resolved gradually (Figure 4).

**Figure 5:**
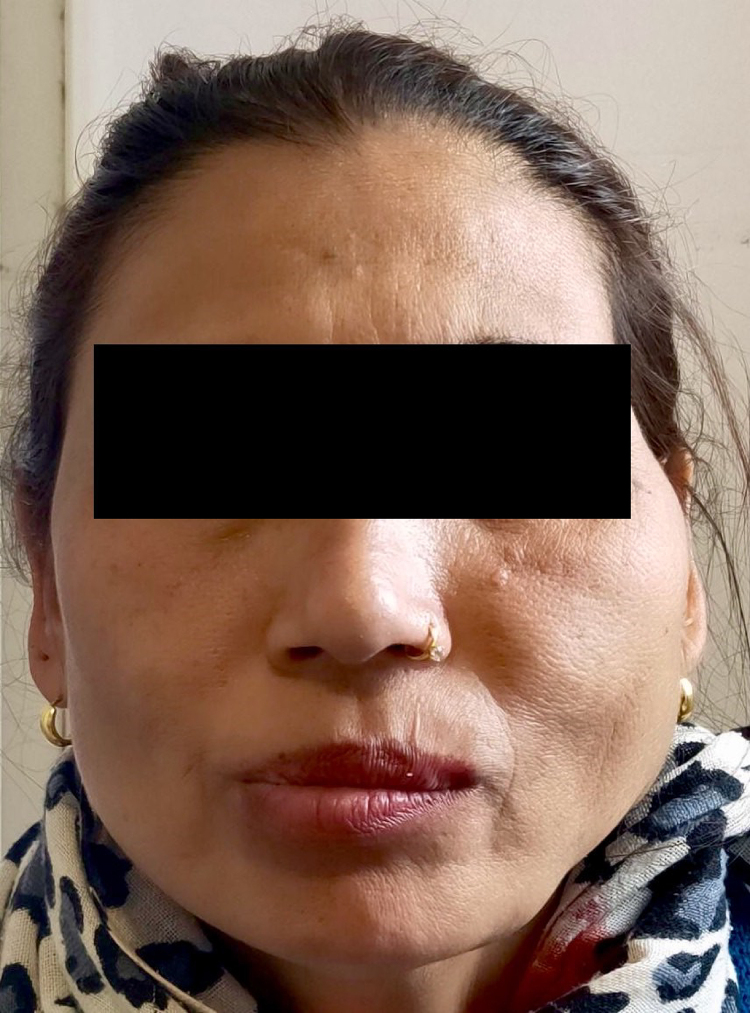
One-year follow-up of the patient shows a marked reduction in swelling

At annual follow-up, there was almost complete resolution of the lesion with no recurrence (Figure 5). Currently, the patient does not have any complaints with chewing and is satisfied with the correction of facial asymmetry.

## Discussion

Revised International Society for the Study of Vascular Anomalies classification divides vascular malformations into two types: simple and combined.^1^ Combined vascular malformation is defined as two or more vascular malformations (capillary, venous, arteriovenous, lymphatic) found in one lesion.^1^

These anomalies most commonly affect the craniofacial regions, are present since birth and enlarge rapidly owing to hormonal changes and traumatic insults.^3^ Clinical features vary with the site, size and duration of formation of these lesions.^2^ They can range from minor symptoms such as a cutaneous stain, pain and tissue overgrowth to life-threatening conditions such as venous hypertension, heart failure and gangrene formation.^3^ Therefore, a thorough clinical evaluation should be carred out with detailed history-taking and clinical examination,^3^ while employing imaging modalities such as Doppler ultrasonography, magnetic resonance imaging (MRI) and/or contrast-enhanced computed tomography to distinguish the type of vascular malformation and the extent into the surrounding structure.^3^

There exist no consensus reporting guidelines or treatment protocols for these lesions, creating intricacy in their management.^3^ Hence, a multidisciplinary approach is mandatory for treating these cases.^3^ This incorporates surgical therapy, embolisation and sclerotherapy performed by vascular surgeons in collaboration with plastic surgeons and interventional radiologists.^3^ In addition, caregivers should aim at correcting the facial disfigurement to meet the patients’ cosmetic concerns, thereby mitigating social stigmatisation.^4^

Lakkasetty *et al* reported a case of multifocal vascular malformation, mostly affecting the craniofacial region, associated with difficulty in chewing, pain and bleeding from the mouth for which MRI was undertaken revealing multiple arteriovenous malformations along with phleboliths.^2^ Contrary to their case, our patient had a solitary malformation in the face with no haemorrhagic manifestation. The lesion was further evaluated with MDCT, which showed combined malformation with arteriovenous and lymphatic components.

A similar case in a child with painful bluish swelling of the right midface was reported by Ashok *et al* and was diagnosed as vascular malformation using MRI angiography.^4^ Unlike in Ashok *et al*, no classical clinical findings of vascular malformation were present in our case. In contrast to embolisation, as undertaken in Ashok *et al*, preoperative use of sclerotherapy and intralesional triamcinolone injection was administered in our case. This helped in effectively reducing the size of the lesion, alongside facilitating resection and improving the outcome. Intraoperative ligation of the feeding vessel with resection of the vascular malformation was followed by the use of sclerotherapy without coil embolisation postoperatively to remove residual intramuscular vascular malformation based on the surgeon’s clinical judgement. Resection of the complete mass would have hampered the patient’s masticating abilities. Kohout *et al* also opted to use sclerotherapy in vascular malformations for devascularisation in cases in which complete resection is not completely possible.^3^

## Conclusions

Combined vascular malformation needs meticulous evaluation with a multidisciplinary approach involving vascular surgeons, radiologists, physiotherapist and oro-maxillofacial surgeons for management. Surgery should aim at removing the lesion, correcting facial disfigurement and preserving crucial functions like mastication.
